# Understanding the Phonetic Characteristics of Speech Under Uncertainty—Implications of the Representation of Linguistic Knowledge in Learning and Processing

**DOI:** 10.3389/fpsyg.2022.754395

**Published:** 2022-04-25

**Authors:** Fabian Tomaschek, Michael Ramscar

**Affiliations:** Quantitative Linguistics Lab, Department of General Linguistics, University of Tübingen, Tübingen, Germany

**Keywords:** linguistic knowledge, discriminative learning, cue-to-outcome structure, morphological structure, phonetic characteristics, reduction, enhancement, context

## Abstract

The uncertainty associated with paradigmatic families has been shown to correlate with their phonetic characteristics in speech, suggesting that representations of complex sublexical relations between words are part of speaker knowledge. To better understand this, recent studies have used two-layer neural network models to examine the way paradigmatic uncertainty emerges in learning. However, to date this work has largely ignored the way choices about the representation of inflectional and grammatical functions (IFS) in models strongly influence what they subsequently learn. To explore the consequences of this, we investigate how representations of IFS in the input-output structures of learning models affect the capacity of uncertainty estimates derived from them to account for phonetic variability in speech. Specifically, we examine whether IFS are best represented as outputs to neural networks (as in previous studies) or as inputs by building models that embody both choices and examining their capacity to account for uncertainty effects in the formant trajectories of word final [ɐ], which in German discriminates around sixty different IFS. Overall, we find that formants are enhanced as the uncertainty associated with IFS decreases. This result dovetails with a growing number of studies of morphological and inflectional families that have shown that enhancement is associated with lower uncertainty in context. Importantly, we also find that in models where IFS serve as inputs—as our theoretical analysis suggests they ought to—its uncertainty measures provide better fits to the empirical variance observed in [ɐ] formants than models where IFS serve as outputs. This supports our suggestion that IFS serve as cognitive cues during speech production, and should be treated as such in modeling. It is also consistent with the idea that when IFS serve as inputs to a learning network. This maintains the distinction between those parts of the network that represent message and those that represent signal. We conclude by describing how maintaining a “signal-message-uncertainty distinction” can allow us to reconcile a range of apparently contradictory findings about the relationship between articulation and uncertainty in context.

## 1. Introduction

The phonetic characteristics of speech signals are highly variable. Separating the variability that is simply noise from that which is informative is central to our understanding of speech. Some parts of this problem have been solved. It is known that variability occurs in relation to coarticulation (e.g., Öhman, 1966; Zsiga, 1992; Magen, 1997), speaking rate (e.g., Lindblom, 1963; Gay, 1978), syllable position (Pouplier and Hoole, 2016), prosody (Mooshammer and Fuchs, 2002; Mücke et al., 2009) and even the idiosyncrasies of speakers (e.g., Tomaschek and Leeman, 2018; Gittelson et al., 2021). By contrast, there is still much debate about the way that representations of linguistic knowledge—and the differing levels of uncertainty associated with this knowledge—serve to co-determine articulation, and in turn the phonetic characteristics of speech. This is especially the case when it comes to the representation of words within inflectional paradigms and the way that the uncertainty associated with different word-forms correlates with fine phonetic detail in the speech signal. Some studies report effects of reduction associated with lower *paradigmatic uncertainty*—mirroring findings within the information theoretic and the *Smooth Signal Redundancy Hypothesis* framework. By contrast, work within the *Paradigmatic Signal Enhancement Hypothesis* framework reports the enhancement of phonetic characteristics (these findings are discussed in detail below).

In what follows, we investigate these effects by addressing the relationship between the uncertainty associated with the inflectional functions of German word-final [ɐ], as in the word *Lehrer* ['le:.ʁɐ] “teacher”, and the phonetic characteristics of [ɐ]. This phone discriminates roughly sixty different grammatical and inflectional functions in German, in morphologically simple and complex words, making it an ideal test bed for this research.

One potential confound in the earliest studies investigating the effects of sublexical relationships on articulation lies in their operationalizations of paradigmatic relations, which were based on theoretically motivated definitions of word-internal structure. To avoid having to make these kinds of assumptions, we follow the approach of Tucker et al. (2019) and Tomaschek et al. (2019) who investigated these phenomena from a discriminative learning perspective. In this approach, which employs a simple neural network trained with an error-driven learning algorithm (widely known as the *delta-rule*), paradigmatic uncertainty is an emergent property within lexical systems, which develops as the individual items it comprises are learned. In doing this, we shall also address some often neglected questions that this approach raises. Psycholinguistic studies using neural networks have typically ignored the way that implementational choices concerning the relationships between inputs and outputs in a network can shape its performance. However, as Bröker and Ramscar (2020) demonstrate, decisions about the input-output structure of computational learning models serve to co-determine what these models actually learn. This in turn affects researchers' interpretations of the performance of models in relation to their theoretical contribution. Accordingly—and in line with the topic of this special issue—a further aim of this work will be the investigation of the kind of input-output structure that is most appropriate for the representation of morphological and inflectional paradigms. Specifically, we shall examine whether inflectional functions of [ɐ] are best characterized as serving as inputs to neural networks or as their outputs, as implemented in Tucker et al. (2019) and Tomaschek et al. (2019).

To analyze the performance of our network models (which we also describe in detail below), we use simulated activations as a measure of the uncertainty associated with each inflectional function. These are regressed against the phonetic characteristics of [ɐ] in order to assess their capacity to predict the phonetic characteristics of the speech signal. We show an enhancement of [ɐ]'s phonetic characteristics associated with lower paradigmatic uncertainty. Critically, we find that when inflectional functions of [ɐ] serve as inputs to the learning network, uncertainty associated with these functions obtained from the network is a better statistical predictor for [ɐ]'s phonetic characteristics than when inflectional functions serve as outputs. Accordingly, the present study contributes to a line of research that investigates how uncertainty affects speech production through a combination of computational modeling of learning and an examination of the predictions of these models for the phonetic characteristics of actual speech (for example Baayen et al., 2019; Tomaschek et al., 2019; Tucker et al., 2019; Stein and Plag, 2021; Schmitz et al., 2021b in the present special issue).

We begin by discussing the empirical and theoretical background of this study, as well as previous work by Tucker et al. (2019) and Tomaschek et al. (2019) that we seek to further examine. We then describe our simulations and analyses before discussing the theoretical and computational implications of our results.

## 2. Background

### 2.1. Phonetic Characteristics and Paradigmatic Probability

It is well-established that phonetic reductions occur in contexts where syntagmatic uncertainty is low. Lower uncertainty has been shown to be associated with shorter words, syllables and segments (Aylett and Turk, 2004; Cohen Priva, 2015) and more centralized vowels (Wright, 2004; Aylett and Turk, 2006; Munson, 2007; Malisz et al., 2018; Brandt et al., 2019). This has been demonstrated by studies that operationalized uncertainty by means of word frequency (Wright, 1979, 2004; Fosler-Lussier and Morgan, 1999; Bybee, 2002), conditional probability (Jurafsky et al., 2001a,b; Aylett and Turk, 2004; Bell et al., 2009), or informativity (Cohen Priva, 2015; Schulz et al., 2016; Malisz et al., 2018; Brandt et al., 2019, 2021). Aylett and Turk (2004, 2006)'s *Smooth Signal Redundancy Hypothesis* explains these *reduction* phenomena from an information theoretic perspective (Shannon, 1948), arguing that the amount of information in the speech signal is balanced against the amount of information conveyed at the syntagmatic level. These systematic findings sparked a line of research that investigated whether equivalent changes in phonetic characteristics can be found when uncertainty is operationalized within other contexts, such as morphological and paradigmatic families.

However, while there is an abundance of evidence showing a systematic relation between uncertainty within these contexts and the phonetic characteristics of speech, when it comes to uncertainty within morphological families, the effects of this relationship seems to run in the *opposite* direction to those reported at the syntagmatic level. Numerous studies have shown lower uncertainty within morphological families to be associated with *enhancement*. This is reflected in longer word durations (Lõo et al., 2018) and consonant durations at compound boundaries (Bell et al., 2019), in longer interfixes in Dutch compounds (Kuperman et al., 2007), in more enhanced articulatory positions in stem vowels of English verbs (Tomaschek et al., 2021), in lower deletion probabilities of the word final [t] in Dutch words (Schuppler et al., 2012) and in Dutch regular past-participles (Hanique and Ernestus, 2011), and in less centralized vowel articulations in Russian verbal suffixes (Cohen, 2015). Kuperman et al. (2007) have proposed the *Paradigmatic Signal Enhancement Hypothesis* to provide a theoretical formalization of these patterns of findings, arguing that phonetic enhancements are a consequence of the greater levels of paradigmatic support that these voicings receive. However, while it might may seem that the findings just discussed appear to contradict one another, it is not entirely clear whether they actually do.

This is because although the studies just described do appear to support the *Paradigmatic Signal Enhancement Hypothesis*, other studies have found an opposite effect, demonstrating an association between lower uncertainty in morphological and paradigmatic families and *reduction*. This is reflected, for example, in higher deletion probability of [t] in derived adverbs (e.g., *swiftly*) (Hay, 2004) and in Dutch irregular past-participles (Hanique and Ernestus, 2011), in shorter [ə] durations in Dutch prefixes (Hanique and Ernestus, 2011), in shorter duration of English prefixes and their consonants (Ben Hedia and Plag, 2017; Plag and Ben Hedia, 2017), and finally, in more centralized [-i] and [-o] when they serve as suffixes in Russian (Cohen, 2015). The different effects associated with paradigmatic uncertainty—enhancement or reduction—emerge independently of the kind of probabilistic measure used to operationalize uncertainty in the domain of morphological and paradigmatic families. That is, regardless of whether paradigmatic uncertainty is operationalized as family size, as word frequency divided by the summed frequency of all the words in a paradigm, or as the frequency of a morphologically complex word divided by its base frequency.

Thus far in this discussion, we have treated the idea of uncertainty in linguistic knowledge as if it is an objective matter of fact. There are, however, good reasons to believe this is not the case. First, because all of the measures used to operationalize the uncertainty associated with different kinds of knowledge are based on theoretical assumptions. Second, because these theoretical assumptions typically disregard the fact that all morphological knowledge is *learned*. Since languages are learned, it necessarily follows that the word-internal structures and distinctions posited by any given theory are unlikely to correspond exactly to the structures and distinctions that have actually been learned by a given speaker at any given point in time.

Tucker et al. (2019) and Tomaschek et al. (2019)'s solution to this problem was to model learning by means of a two-layer neural network that was trained with an error-driven learning rule (the delta rule Rescorla and Wagner (1972); Rumelhart and McCelland (1987), provided by the Naive Discriminative Learner package in R, Arppe et al., 2018). If trained in a naive way, the neural network does not explicitly embody the structures of linguistic knowledge that are typically assumed in psycho-linguistic theories. Rather, the model's representation of these structures emerges in bottom-up fashion, as a result of training the network. As a consequence, knowledge in the model is represented by the distribution of its connection weights such that “morphological structure” emerges gradually, in gradient fashion, as the model is trained^1^.

Tucker et al. (2019) and Tomaschek et al. (2019) used network measures to operationalize uncertainty within a morphological paradigm. The results of these studies showed lower uncertainty to be associated with longer stem vowel duration in regular and irregular English verbs and longer duration of word final [s] that encodes multiple inflectional functions (plural noun, genitive, second person singular verbs, etc.). Accordingly, these results provided evidence to corroborate the claim that phonetic enhancement is associated with lower paradigmatic uncertainty.

Because the present study builds on the work by Tucker et al. (2019) and Tomaschek et al. (2019), we shall need to discuss their models and input-output structures in detail. However, before we can do so, it is first important that we flesh out the theoretical background to this work. This is because, as we noted above, we do not only aim to examine the relation between paradigmatic uncertainty and articulation here. Our goal is also to provide a theoretical examination of the way that the various factors that contribute and provide evidence for these effects are best represented in neural network models (see also Bröker and Ramscar, 2020; Ramscar, 2021a).

Accordingly, we shall begin by discussing how previous computational models of speech production have addressed these issues, and how they were used to make predictions about the phonetic characteristics of speech. Then, since both Tucker et al. (2019) and Tomaschek et al. (2019) are rooted in the theory of *discriminative learning*, a cognitive theory of how language (and actually any kind of behavior) is learned (Ramscar and Yarlett, 2007; Ramscar et al., 2010, 2013b; Ramscar, 2019, 2021b), we shall examine the constraints that this theory imposes on the way the input-output structure of models is configured.

### 2.2. Computational Models of Speech Production

Researchers in the twentieth century collected a great deal of information in the form of speech errors and data from controlled psycho-linguistic experiments. This information then informed theoretical speculations about the nature of the speech production process (e.g., Fromkin, 1971; Levelt et al., 1999). While these psycho-linguistic theories are useful at a general level, they are subject to the standard limitations of all verbal theories. One of the limitations is that they are open to interpretation and that they are often vague when it comes to the specific details of processing. Computational models, such as those presented by Dell (1986) and Roelofs (1997) ameliorate these problems of vagueness. These models force language researchers to make definitive commitments regarding the detailed structure of processes, regarding the kinds of algorithms involved and, of importance to the present study, regarding the structure of the representations that are required to model speech production. In return for these commitments, researchers are not only able to eliminate some of the vaguenesses in theory, they are also able to obtain quantitatively testable predictions. While most research on computational models of speech production has focused on the structure of models at an algorithmic level, the structure of the input and output to/from these models has been largely taken for granted. However, the performance of computational models does not only depend on their individual architectures and algorithms. The representation of knowledge in the model can also have a critical bearing on its behavior. That is, the structure of its inputs (on which its predictions are based) and its outputs (what it predicts) can systematically change how a model performs. Indeed, as Bröker and Ramscar (2020) recently demonstrated, depending on the representational assumptions made, different models of the same empirical result can provide support for psycho-linguistic theories that make opposing claims about the nature of learning and processing.

**Figure 1:**
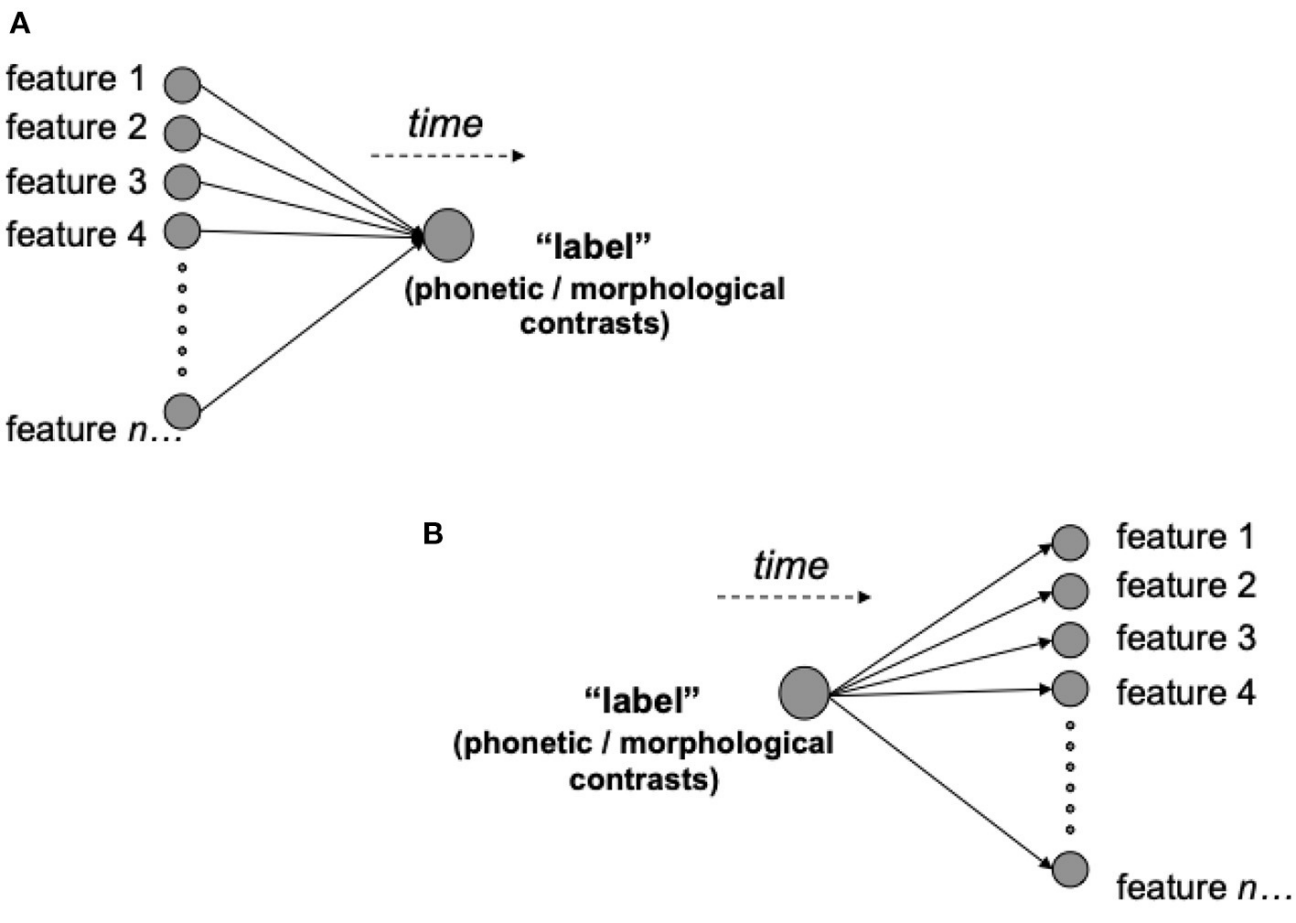
The possible predictive relationships labels (in morphological terms, series of words and affixes) can enter into with the other features of the world (or other elements of a code). A feature-to-label relationship **(A)** will facilitate cue competition between features, and the abstraction of the informative dimensions that predict morphological contrasts (e.g., nouns and plural affixes) in learning. By contrast, a label-to-feature relationship **(B)** will be constrained to simply learning the probability of each feature given the label.

The relation between input-output structures and the subsequent interpretation of performance become further apparent when we consider computational models such as WEAVER++ (e.g., Roelofs, 1997) or the Spreading-Activation Theory of Retrieval (Dell, 1986; Dell et al., 2007, and follow-up models). These models use a network framework that reflects a common conceptualization of speech production in psycho-linguistics, assuming it to be a sequential, transformational process. At the highest level, the production of spoken words is initiated by information that represents the semantics of the words to be uttered. These in turn activate discrete information at lower levels of processing such as morphemes, syllables, and finally phonemes^2^. In terms of the representation of linguistic knowledge, this means that the complexity of information within these models fans out into more and more fine grained units. This situation is illustrated in Figure 1B where “label” can be taken as a placeholder for any kind of higher level units of information—e.g., inflectional functions or morphological contrast—and “feature 1”, “feature 2”, etc. can be regarded as a placeholder for lower level units—e.g., phones. This raises a question: How reasonable is this flow of information from the perspective of learning theory? We address this in the next section.

### 2.3. Linear Order and Discriminative Learning

It seems clear that where systematic patterns of variance in production have been seen to relate to morphological and paradigmatic structure, these effects must be a product of what speakers have learned. The mechanisms that support this learning thus offer an obvious source of explanation for the patterns of behavior observed. While different kinds of mechanisms have been proposed for language learning (see e.g., Ellis, 2006), research has revealed that the majority of human (and animal) learning mechanisms are based on prediction and prediction-error, i.e., error-driven learning (O'Doherty et al., 2003; Schultz, 2006).

Rescorla and Wagner (1972)'s implementation of the delta rule defines a simple error-driven learning algorithm that is often used in psychological research, and was used by Tucker et al. (2019) and Tomaschek et al. (2019) to train their two-layer networks (a detailed description is provided in their Appendix)^3^. Its algorithm implements a systematic learning process that aims to produce a set of mappings that best discriminate the informative, predictive relationships between a set of inputs and a set of outputs given a training schedule. Because of this, Ramscar et al. (2010) suggest that from a computational perspective the algorithm is best understood as describing a discriminative learning mechanism^4^.

Because prediction is a time-sensitive process, the order in which experiences occur is a strong determinant of the kind information that can be learned about cue-outcome relationships through error-driven learning (Ramscar et al., 2010; Arnon and Ramscar, 2012; Hoppe et al., 2020; Vujovic et al., 2021). Speech comprises an ordered series of gestures. These yield an ordered series of phonetic contrasts (Nixon and Tomaschek, 2020) that represent an ordered series of linguistic events (Dell et al., 1997; Grodner and Gibson, 2005). Given that it seems clear that language is learned through an error-driven mechanisms it follows that speech production is likely to be particularly sensitive to these sequential/time-sensitive effects.

However, although speech is clearly ordered, in its use in communication it supports “displaced reference” (Hockett and Hockett, 1960). That is, it allows for reference to things that are not present in the here and now. One consequence of this is that the constraints that are imposed by predictive relationships in language use are not always obvious. This is especially the case when it comes to the relations between form and meaning in linguistic morphology (Ramscar et al., 2010; Ramscar, 2013; see also Ramscar, 2021a for a general review of this issue in relation to morphology).

To explain these constraints, it is first important to note that because prediction and prediction error modulate the values of cue-outcome relationships, these values are not determined by simple co-occurrence. Rather, when multiple cues to an outcome are present, a given cue's value will depend on a competitive process that weighs the informativity of each cue in relation to the current uncertainty of a learner. This situation is illustrated in Figure 1A, where multiple present features compete for the prediction of an outcome or a label. Informativity thus takes into account both co-occurrences between a cue and an outcome and the non-occurrence of the outcome given the cue. Because uncertainty is finite, more informative cues gain value at the expense of less informative cues. In other words, cues compete for predictive value, a process that leads to the discovery of reliable cues through the discriminatory weakening and elimination of other cues (Ramscar et al., 2010; Nixon, 2020).

While this mechanism is simple in principle, in practice it is an extremely efficient method for extracting predictive structures. For example, in English morphology, plurality is typically marked on nouns by a final sibilant /s/ (whose voicing depends on phonetic context).

The existence of this predictable regularity has implications for the informativity of cues about inflectional structures. Someone learning to predict the form of English nouns will be presented with a large number of cues to the wide range of articulatory events that English nouns comprise. Most of the plural nouns that children encounter will tend to provide evidence for the highly informative cue-outcome relationship between plurality and the presence of a final sibilant at the end of the noun's form. Because of this, it follows that once children have begun to learn the cues to nouns, the relationship between plurality and a final sibilant at the end of nouns can be expected to be reliably learned. However, because this relationship is not informative about the subset of irregular plurals, children will have to learn to ignore this cue in irregular contexts, and learn the more specific cues to these nouns instead. It follows from this that until children have learned to ignore the more general cue to regular plurals, the intermediate representation they acquired may cause them to over-regularize irregulars (Ramscar and Yarlett, 2007; Ramscar and Dye, 2009; Ramscar et al., 2013b). In the same way that children learn to ignore the erroneous cues to irregulars, they will also learn that the other, less informative cues associated with regular plurals should also either be ignored, or associated with other parts of the signal (Ramscar et al., 2010, 2013a). Accordingly, as speech unfolds in time, similar forms of this process will allow for the many abstract features associated with verbs and their suffixes (e.g., tense, aspect etc.) to be learned and extracted in much the same way.

In addition, because learning happens in time, and because the events signaled in speech occur serially, it follows that linguistic regularities (or “units”) can serve as both cues and outcomes in learning. For example, in the sentence ‘The girl plays football', “girl” predicts “plays” which in turn predicts “football”. It thus follows that, when all of these considerations are taken together, determining exactly what counts as a cue and what counts as an outcome in speech production is not always obvious. Moreover, when it comes to modeling, these matters will often be determined by the specific goals of the model.

### 2.4. Cue-to-Outcome Structure in Speech Production and Implications for Input-Output Structures

With cue competition, prediction and prediction error in mind, we can conceptualize speech production and articulation from the perspective of discriminative learning. As we discussed earlier, in existing psycho-linguistic theories of speech production, semantics, inflectional and morpho-syntactic information should serve as cues for articulation. In addition to these high level sources of information, there is evidence that articulation is also driven by articulatory, sensory and acoustic targets (“articulatory target cues”, cf. Hickok, 2014; Guenther, 2016). From a discriminative perspective, all these cues will compete simultaneously for informativity about the executed articulatory gestures during learning. As a consequence, it follows that during production, these cues will serve to activate the execution of articulatory gestures. Note that we do not make any statements about the size of gestural chunks. Following Guenther (2016), we assume that their size can range between a single phone, and sequences of multiple phones. Moreover, even the size of the “same chunk” might vary, depending, for example, on the amount of practice a particular speaker has with them (see Tomaschek et al., 2018a,c, 2020; Saito et al., 2020a,b, for electromagnetic articulography and ultrasound studies on practice).

It thus follows from the above that when it comes to the computational modeling of speech, it is these semantic, morpho-syntactic, inflectional and articulatory target cues that should serve as the *inputs* to neural network learning models. In the same vein, the articulatory gestures that will be activated by these cues should serve as the *outputs* of these models.

However, Tucker et al. (2019) and Tomaschek et al. (2019) did not employ this input-output structure to train the networks described earlier. Rather, following the approach taken by Baayen et al. (e.g., 2011, 2016b), in the model of Tucker et al. (2019) the target gestures served as the only inputs—reflected by diphones of words in the Buckeye Corpus (Pitt et al., 2007). The outputs of the model then consisted of the tense of the verbs under investigation, in addition to inflected word forms. This meant that, from the perspective of our analysis above, the outputs of this models contained information that actually serves as inputs when speakers learn to articulate inflections.

Tomaschek et al. (2019) followed Tucker et al. (2019)'s example regarding the input-output structure, but extended the input to a five-word window around the targeted word in the Buckeye corpus. From this five-word window, two kinds of inputs for the network were extracted. First, diphones from all words that served as an approximation of acoustic and sensory targets that serve to initiate articulation in models of speech production (Hickok, 2014; Guenther, 2016). Second, the word forms preceding and following the target word. These word form inputs were assumed to capture the target word's semantic embedding—in the same way that studies of distributional semantics counted the number of co-occurrences between words within a specific context (Lund and Burgess, 1996; Landauer et al., 1997; Shaoul and Westbury, 2010; Mikolov et al., 2013), and in the same way that studies within the framework of “naive discriminative learning” used word forms to discriminate word meanings (Baayen et al., 2016a,b). As outcomes, the inflectional functions encoded by word final [-s] in English were used. In summary, this meant that the input-output structure provided to the neural networks in both of these studies did not reflect the cue-to-outcome structure that actually seems appropriate to speech production. Instead, some of the information that was represented as outputs in these models actually appears to serve as inputs when production is analyzed from a learning perspective. With this theoretical and empirical background in mind, we turn to the specific aims of the present study.

### 2.5. The Present Study

The general aims of the present study are: (a) to train a two-layer neural network with an input-output structure that contains the inflectional information relevant to German word final [ɐ]; (b) to use the resulting network measures to predict the phonetic characteristics of [ɐ]. Since findings are contradictory regarding the relationship between uncertainty within the morphological and paradigmatic context and phonetic characteristics, it followed that at the outset, the expected direction of this relationship was unclear.

The network measures might be associated with enhancement, as predicted by the *Paradigmatic Signal Enhancement Hypothesis* (Kuperman et al., 2007) and demonstrated by previous studies using two-layer network models (Tomaschek et al., 2019; Tucker et al., 2019); or they might be associated with reduction, as predicted by the *Smooth Signal Redundancy Hypothesis* (Aylett and Turk, 2004; Cohen Priva, 2015). Accordingly, another aim of this study was to empirically determine which of these hypotheses is supported by a model that accurately captures the dynamics of morphological learning.

Accordingly, the study also aimed to compare the performance of a two-layer learning network employing the input-output structure used by Tucker et al. (2019) and Tomaschek et al. (2019)—where inflectional functions served as outputs—to one in which these functions were represented appropriately: as inputs to the output gestures that represent their realization in speech. We will refer to these learning networks as the *functional output network* and *functional input network*, respectively. We expected that measures extracted from the *functional input network* would be a better predictor of phonetic characteristics than measures computed on the basis of the *functional output network*.

## 3. Methods

### 3.1. Material

The materials for the present study were extracted from the Karl-Eberhards-Corpus of spontaneously spoken southern German (KEC, Arnold and Tomaschek, 2016). The KEC contains recordings of two acquainted speakers having a spontaneous conversation for 1 h about a topic of their own choosing. Speakers were seated in two separate recording booths and their audio signal was recorded on individual channels so that the audio of each speaker can be analyzed without the interference from the other. The KEC contains manually corrected word boundary annotations and forced-aligned segment annotations obtained using the Rapp forced aligner (version 2015, Rapp, 1995).

The corpus contains a total of roughly 23,100 word tokens (1,360 types) that contain a word-final [ɐ]. To make sure the segment annotations are correct, we manually corrected all [ɐ] instances in the corpus for which the aligner provided an annotation. We excluded all instances for which the aligner failed to perform the annotation. This was the case when there was too big a mismatch between the expected and actual duration of the word. In these cases, it was also very hard to annotate the [ɐ] as it was unclear, due to the strong reduction of the [ɐ]-bearing word, where to place segment boundaries. We also excluded the article *der* from the analysis since its annotation is complicated: its pronunciation ranges between [de:ɐ], [dɛ:ɐ], [dɐ], etc. and it is at times unclear at what point the boundary between the two vowels, if present, should be made.

The final data set for the analysis in the present study contained 10,320 word tokens (870 types). It contained 4,944 content words (e.g., nouns, adjectives), 4,463 morphologically simple function words (e.g., adverbs) and 913 morphologically complex function words (e.g., demonstrative pronouns).

The inflectional functions encoded by [ɐ] in these words was manually classified. In total, 60 inflectional functions were obtained, based on combinations of grammatical functions (nouns, articles, pronouns, etc.), numerus (singular, plural), gender (feminine, masculine, neuter) and case (nominative, genitive, dative, accusative). A list of all functions can be found in the Supplementary Material (https://osf.io/8jf5s/).

As a measure of spectral characteristics, we investigated the time courses of the first and second formant (F1, F2). We used the LPC algorithm provided by Praat (Boersma and Weenink, 2015, standard settings) to compute the time courses of F1 and F2 in each vowel. For analysis, we excluded vowels shorter than 0.018 s (log = −4) due to sparse data. In addition, we excluded formant measurements for which F1 was outside a range between 250 and 1,000 Hz, and F2 was outside a range between 1,000 and 2,000 Hz. As a result of this exclusion, additional 112 word tokens were excluded, yielding a data set of 11,018 word tokens (871 types) with word final [ɐ] for the analysis. Words with word final [ɐ] will be called *[*ɐ*]-word* from now on. In order for higher tongue positions to be associated with higher F1 values, thus making F1 frequencies straightforwardly interpretable, F1 frequencies were inverted by being multiplied by −1. Prior to analysis, formant frequencies were centered and normalized by speaker.

### 3.2. Assessing Uncertainty

In this section, we discuss the details of the input-output structures discussed in the introduction and how we implemented them in the *functional output network* and the *functional input network*. We used the entire KEC to construct the learning events on the basis of which we trained the two network models. Learning was simulated using the Rescorla and Wagner (1972)'s delta-rule [as implemented in the *Naive Discriminative Learner* package 2, Shaoul et al. (2014)]. An explanation of the delta-rule can be found in the Appendix of Tomaschek et al. (2019). As noted above, apart from information about inflectional function, several other sources of information serve as cues to speech production. To operationalize these other cues, we followed Tomaschek et al. (2019)'s approach. Accordingly, both models described below used cues derived from a five-word sliding window that iterated across all learning events. Keeping the rest of the cue structure consistent across the models (and studies) ensured comparability between both the two models implemented here and the previous studies.

#### 3.2.1. Knowledge Representation in the Functional Output Network

The input-output structure used to train the *functional output network* was essentially the same as that employed by Tomaschek et al. (2019). Inputs consisted of the word forms preceding and following the target word in the five-word sliding window. The target word itself never served as an input to avoid direct mappings between inputs and outputs. In addition, inputs contained the diphones of all words in the sliding window including the target word. Diphones were based on the phonetic transcription provided by the Rapp forced aligner used to annotate the corpus (Rapp, 1995).

As in the Tucker et al. and Tomaschek et al. studies, the outcomes in the *functional output network* were the morphological and inflectional functions of the [ɐ]-words. Recall that the network iterated across all word events in the KEC corpus. This means that it also encountered numerous words that did not have word-final [ɐ], and accordingly no inflectional function of interest. In this case, a simple place holder was used to ensure cue competition. To summarize, the *functional output network* was trained to predict inflectional functions of [ɐ]-word on the basis of word and diphone cues.

To obtain a predictor of phonetic characteristics of [ɐ], we computed *functional output activation* on the basis of the trained network. The measure can be regarded as a measure of the uncertainty about the inflectional functions that emerges within the five-word sliding window. *Functional output activation* was computed by summing the weights between all word and diphone inputs in the five-word window around the [ɐ]-word and the inflectional functions of the target word.

#### 3.2.2. Knowledge Representation in the Functional Input Network

The input-output structure in the *functional input network* followed the logic of our analysis in the introduction, where we argued that inflectional functions are learned to serve as cues in speech production and hence should actually serve as inputs to the learning process simulated in the network (Ramscar et al., 2013b; Ramscar, 2021a, see also). Also consistent with this analysis, the outcome of the articulation process, [ɐ], functioned as the output of the network. Accordingly, in addition to diphones and words within the five-word window (the same as in the previous structure), we used the inflectional functions of the words with final [ɐ] as inputs. The output of the network was [ɐ], whenever it was in word-final position of [ɐ]-bearing words. In line with the interpretation by Tomaschek et al. (2019), we regard the outcome [ɐ] to function as an abstract placeholder for potential articulatory gestures representing the articulation of [ɐ] in context. In other words, this network was trained to predict the occurrence of [ɐ] on the basis of word forms, diphones and the inflectional functions. To ensure cue competition, we also used the word forms of the target words in the center of the sliding window as outputs. As a predictor of phonetic characteristics, we computed *functional input activation* by summing the weights between all word, diphone and inflectional function inputs in the five-word window and the [ɐ] output. An introduction to training such a two-layer network and coding the calculation of activations can be found in Tomaschek (2020).

#### 3.2.3. Example

To explain the way training proceeded in the two models, consider the following sentence: *Das ist dieser gro*β*e Mann* “This is the big man”. In the *functional output network*, the word inputs in the five-word sliding window centered on *dieser* “this” were das ist dieser groβe mann (we ignored major case). The acoustic diphone inputs in this windows are #d da as sI Is st td di iz z5 5g gr ro os s@ @m ma an n#, with # representing boundary cues. The outputs would be the combination of grammatical and inflectional functions of *dieser*: Demonstrativpronomen Maskulin Nominativ “demonstrative pronoun masculine nominative”. Note that grammatical and inflectional functions were used as separate entries and hence, each of them served as an individual output in a learning event (called multiple-hot encoding in the machine learning community). In the *functional input network*, the inputs in the five-word sliding window centered on *dieser* “this” are the words das ist groβe mann, the acoustic diphones #d da as sI Is st td di iz z5 5g gr ro os s@ @m ma an n#, and the inflection functions Demonstrativpronomen Maskulin Nominativ (multiple-hot encoding). The articulated forms such as *dieser ER*, including a “gestural placeholder” representing the [ɐ]-gesture, served as outputs.

## 4. Analysis and Results

### 4.1. Statistical Analysis of Formant Trajectories

#### 4.1.1. Creating a Baseline Statistical Model

In this section, we describe our statistical approach to analyzing the time course of F1 and F2. We employed generalized additive mixed models (GAMM in the mgcv package, Hastie and Tibshirani, 1990; Wood, 2006, 2011) to investigate how the time course of F1 and F2 in [ɐ] was co-determined by uncertainty in the two models. GAMM uses spline-based smoothing functions to model non-linear functional relations between a response and one or more covariates, modeling wiggly curves using spline smooths as well as wiggly (hyper)surfaces using tensor product smooths (see Wieling et al., 2016; Baayen and Linke, 2020, for an introduction to spline smooths and their use). All model comparisons (and visualization) reported in the following paragraphs were performed with the help of functions provided by the *itsadug* package (van Rij et al., 2015). All analyses can be found in the Supplementary Material.

We constructed a model that contained a smooth “s()” for *time* to model the time course of F1 and F2. Time contained the time points at which formant frequencies were measured. Since vowels vary in duration, time points were normalized to a [0, 1] interval, with 0 linked to vowel onset and 1 to vowel offset. We fitted F1 and F2 simultaneously in one model. Accordingly, we needed a predictor to differentiate between the shapes of F1 and F2 trajectories using a factorial predictor *dimension* with the levels F1 and F2. This predictor interacted with the smooth for *time*. To control for speaker dependent formant trajectories, we fitted by-speaker random factor smooths for time, i.e., the non-linear equivalent of a combination between random intercepts and random slopes from standard mixed-effects regression.

The inclusion of words as random effects caused high concurvity in our models^5^. Accordingly, following the suggestion presented in Baayen and Linke (2020), we did not include words as an random effect. Instead, we controlled for effects of coarticulation with the context by fitting by-place-of-articulation random factor smooths for time for the preceding and for the following segment. To allow random factor smooths to vary depending on dimension, all by-factor smooths included an interaction with *dimension* (F1/F2). We controlled for autocorrelation among residuals using the rho parameter (ρ = 0.8).

In a bottom-up fitting procedure, we tested whether the inclusion of additional predictors improved the model fit. The first additional predictor we tested was *vowel duration*, log-transformed to obtain normally distributed values. *Vowel duration* served as a control variable as it accounted for undershoot and overshoot associated with temporal variation (Gay, 1978). The inclusion of *vowel duration* as a main effect interacting with *dimension* significantly improved model fit (Δ ML = −1106, Δ edf = +4, *p* < 0.0001). Allowing *vowel duration* to interact with *time* and *dimension* (by means of a tensor product smooth “te()”) further improved model fit (Δ ML = −845, Δ edf = +6, *p* < 0.0001). The tensor thus accounts for systematic changes in the shape of the trajectory as a function of *vowel duration*.

German word-final [ɐ] discriminates inflectional and grammatical function in content words (e.g., nouns, adjectives), morphologically complex function words (e.g., demonstrative pronouns) and morphologically simple function words (e.g., adverbs). Numerous studies have reported that higher level information such as inflectional function (Plag et al., 2017; Seyfarth et al., 2018; Schmitz et al., 2021a) or pragmatic function (Drager, 2011; Podlubny et al., 2015) correlate with phonetic characteristics. Similar effects have been demonstrated for word class (e.g., Johnson, 2004; Bell et al., 2009; Fuchs, 2016; Lohmann, 2018; Linke and Ramscar, 2020), for which also processing differences during perception (Neville et al., 1992; Pulvermüller, 1999; Brusini et al., 2017) and production (Fox et al., 2009; Juste et al., 2012) have been demonstrated. Given these systematic differences in perception and production due to higher level information, especially those for word class, we also expect [ɐ] to vary with word class.

This prediction was tested with the predictor *word class*, allowing for potential differences in formant trajectories depending on content words, morphologically complex function words and morphologically simple function words. In order to allow formant trajectories to vary independently in the two dimensions F1 and F2 as well as *word class*, we constructed the factorial predictor “dimension-by-class” (*dbc*) with six levels: one level for each of the six combinations of *dimension* by *word class*. The inclusion of *dbc* as a main effect significantly improved model fit (Δ ML = −405, Δ edf = +4, *p* < 0.0001), as was the case when it was allowed to interact with the *time* by *vowel duration* tensor (Δ ML = −736, Δ edf = +20, *p* < 0.0001). We also tested whether the three levels in *word class* were indeed necessary. We accomplished this by collapsing two levels and refitting the model (e.g., morphologically simple and complex function words were collapsed into one level, and so forth). Collapsing two levels never yielded a better model fit than using *word class* with the three levels. Accordingly, it appears that [ɐ] does indeed vary systematically depending on *word class*. This conclusion is supported by the visualization of the formant trajectories, which are further discussed below. We shall consider this our baseline model, whose formula is illustrated below (with POA = place of articulation):


m0 = formant frequency ~ dbc
+ te(time, vowel duration by = dbc)
+ s(time, speaker, bs=“fs”, m = 1, by =
dimension)
+ s(time, preceding POA, bs=“fs”, m = 1,
by = dimension)
+ s(time, following POA, bs=“fs”, m = 1,
by = dimension)


(The random effects structure, indicated by bs=“fs”, was the same in all models which is why we will not display it anymore in the following formulas).

#### 4.1.2. Testing Activations

In the next analytic stage, we tested the degree to which the inclusion of *functional output activation* and *functional input activation* improved the model fit. The following formula illustrates the model (where *activation* represents both kinds of *activation*):


m1 = formant frequency ~ dbc
+ te(time, vowel duration by = dbc)
+ s(activation, by = dimension)


The question thus arises of whether there are also systematic differences of *activation* depending on *word class*. The following model tested this interaction between *activation* and *dbc*.


m2 = formant frequency ~ dbc
+ te(time, vowel duration by = dbc)
+ s(activation, by = dbc)


We also tested whether the shape of the trajectory was modulated by *activation*. This was accomplished by fitting an interaction between *time* and *activation* and *dimension* using a partial tensor product smooth “ti()”^6^:


m3 = formant frequency ~ dbc
+ te(time, vowel duration, by = dbc)
+ s(activation, by = dbc)
+ ti(time, network measure, by =
dimension)


The final model tested to what degree both the intercept and the shape of the formant trajectories varied in relation to *activation* and *dbc*:


m4 = formant frequency ~ dbc
+ te(time, vowel duration, by = dbc)
+ s(activation, by = dbc)
+ ti(time, network measure, by = dbc)


**Figure 2:**
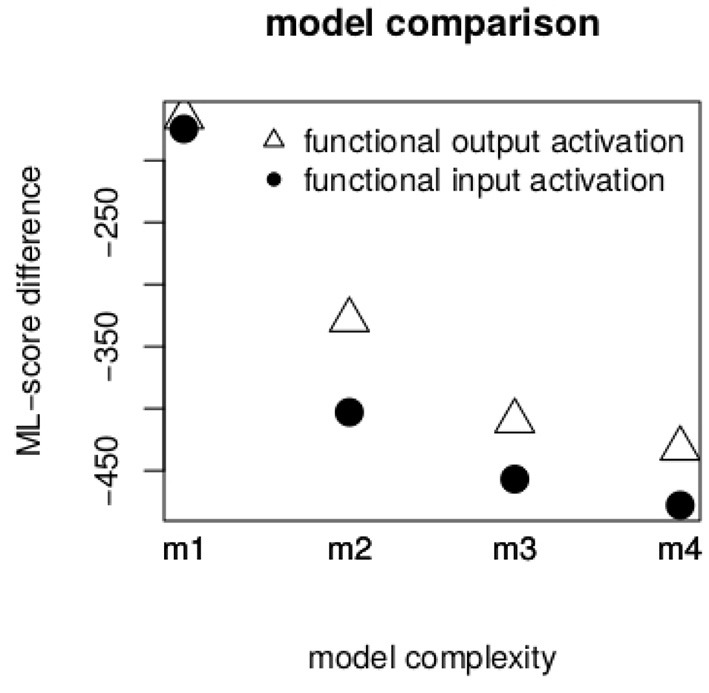
ML-score difference between model m0 and models m1 to m4. The larger the difference, the better the model's goodness of fit.

Figure 2 illustrates the difference in ML-scores between our baseline model *m0* and models *m1* to *m4*. The inclusion of both types of activation improved model fit, as can be seen by means of the large negative ML-score difference for model *m1*. Nevertheless, there was no large difference between the gam model containing *functional output activation* (triangles) and the one containing *functional input activation* (circles) (indeed the difference in ML-score between models with the two types was only 1.5). The goodness of fit depending on the two types of activation changed in more complicated models. In models *m2* to *m4, functional input activation* provided systematically better model fits, as indicated by larger difference in ML-score to *m0*. In other words, a network that was trained to predict the articulatory gesture of [ɐ] on the basis of semantic, phonological and inflectional functions provided better predictions about [ɐ]'s phonetic characteristics than a network trained to predict the inflectional function itself. We also tested to what degree the inflectional function in the input structure is necessary. We found that activations computed on the basis of network trained without inflectional functions as inputs provided a significantly worse model fit than *functional input activation* (on average, they had an ML-score lower by 200). Accordingly, we regard inflectional functions to be necessary in the input structure (model comparisons can be found in the Supplementary Materials).

An inspection of concurvity indicated that the smooths and tensor product smooths for both types of *activation* for morphologically simple function words suffered from high concurvity. Further inspection indicated that this problem was alleviated when individual models were fitted for each level of *word class*. Since the significant interaction with word class (by means of *dbc*) indicated that formant trajectories differ systematically between word classes, fitting individual models for each *word class* was fully supported. Accordingly, below we report the results for models in which formant trajectories were fitted for each of the three levels of *word class* individually. Once models were obtained, data points with residuals larger than 2.5 standard deviations away from the mean were excluded and models were refitted. The following formula illustrates the final model structure:


    m.final = formant frequency ~ dimension
+ te(time, vowel duration, by = dimension)
+ s(activation, by = dimension)
+ ti(time, activation, by = dimension)


### 4.2. Modulation of Formant Trajectories

#### 4.2.1. Summaries

Even though *functional output activation* performed worse than *functional input activation*, we will report the estimated trajectories for both of them to allow for a direct comparison. Summaries of all the statistical models indicated that all the tensor product smooths for the *time* by *vowel duration* in both dimensions (F1, F2) were significant (*p* < 0.001) in all statistical models for all activation types. The same result was found for random factor smooths for participants and for place of articulation of the preceding and following vowel. Since these effects are not of primary interest for the present study, and the summaries use up a lot of space, we provide their summaries only in the Supplementary Material. Here, we report the summaries for the effect of interest, *functional input activation* and *functional output activation*. Table 1 illustrates that all but one smooth and tensor terms for *functional input activation* are significant. Only the partial tensor in the F1 dimension in the model fitting morphologically simple function words failed to be significant. Accordingly, the amplitude of the F1 time course was not significantly modulated. A similar result can be see for *functional output activation*. Here, only the partial tensor product smooth for F1 in morphologically complex function words failed to be significant.

**Table 1:** Summary of the statistical models using functional input activation and functional output activation as a predictor of formant trajectories.

	**edf**	**Ref.df**	***F*-value**	***p*-value**
**FUNCTIONAL INPUT ACTIVATION**				
**Complex function words**				
s(functional input activation):dimension = F1	3.7482	3.9577	39.0716	<0.0001
s(functional input activation):dimension = F2	3.2589	3.7180	44.7998	<0.0001
ti(time,functional input activation):dimension = F1	7.6804	9.7289	2.3730	0.0079
ti(time,functional input activation):dimension = F2	4.6737	5.8764	4.3388	0.0002
**Content words**				
s(functional input activation):dimension = F1	3.3729	3.7829	10.0274	<0.0001
s(functional input activation):dimension = F2	3.8460	3.9845	94.0980	<0.0001
ti(time,functional input activation):dimension = F1	10.4838	12.7473	5.2548	<0.0001
ti(time,functional input activation):dimension = F2	7.7378	9.4625	14.1532	<0.0001
**Simple function words**				
s(functional input activation):dimension = F1	3.8933	3.9921	20.9012	<0.0001
s(functional input activation):dimension = F2	3.7390	3.9562	27.3247	<0.0001
ti(time,functional input activation):dimension = F1	7.3833	9.7127	1.4650	0.1497
ti(time,functional input activation):dimension = F2	10.8514	12.8554	4.6229	<0.0001
**FUNCTIONAL OUTPUT ACTIVATION**				
**Complex function words**				
s(functional output activation):dimension = F1	1.0020	1.0038	115.2282	<0.0001
s(functional output activation):dimension = F2	3.8720	3.9862	12.4216	<0.0001
ti(time,functional output activation):dimension = F1	4.6471	6.5973	0.5412	0.7934
ti(time,functional output activation):dimension = F2	3.6281	4.2364	6.7708	<0.0001
**Content words**				
s(functional output activation):dimension = F1	3.7248	3.9528	5.1275	0.0011
s(functional output activation):dimension = F2	3.9479	3.9976	106.9967	<0.0001
ti(time,functional output activation):dimension = F1	9.6920	12.3538	3.8719	<0.0001
ti(time,functional output activation):dimension = F2	9.9943	12.3734	8.4570	<0.0001
**Simple function words**				
s(functional output activation):dimension = F1	3.2277	3.6812	21.2965	<0.0001
s(functional output activation):dimension = F2	3.9082	3.9942	39.8523	<0.0001
ti(time,functional output activation):dimension = F1	8.0654	9.6352	8.6645	<0.0001
ti(time,functional output activation):dimension = F2	4.9361	6.8905	3.0888	0.0027

*Summaries of control variables and random effect structure can be found in the Supplementary Material*.

#### 4.2.2. Modulation of Formant Trajectory

**Figure 3:**
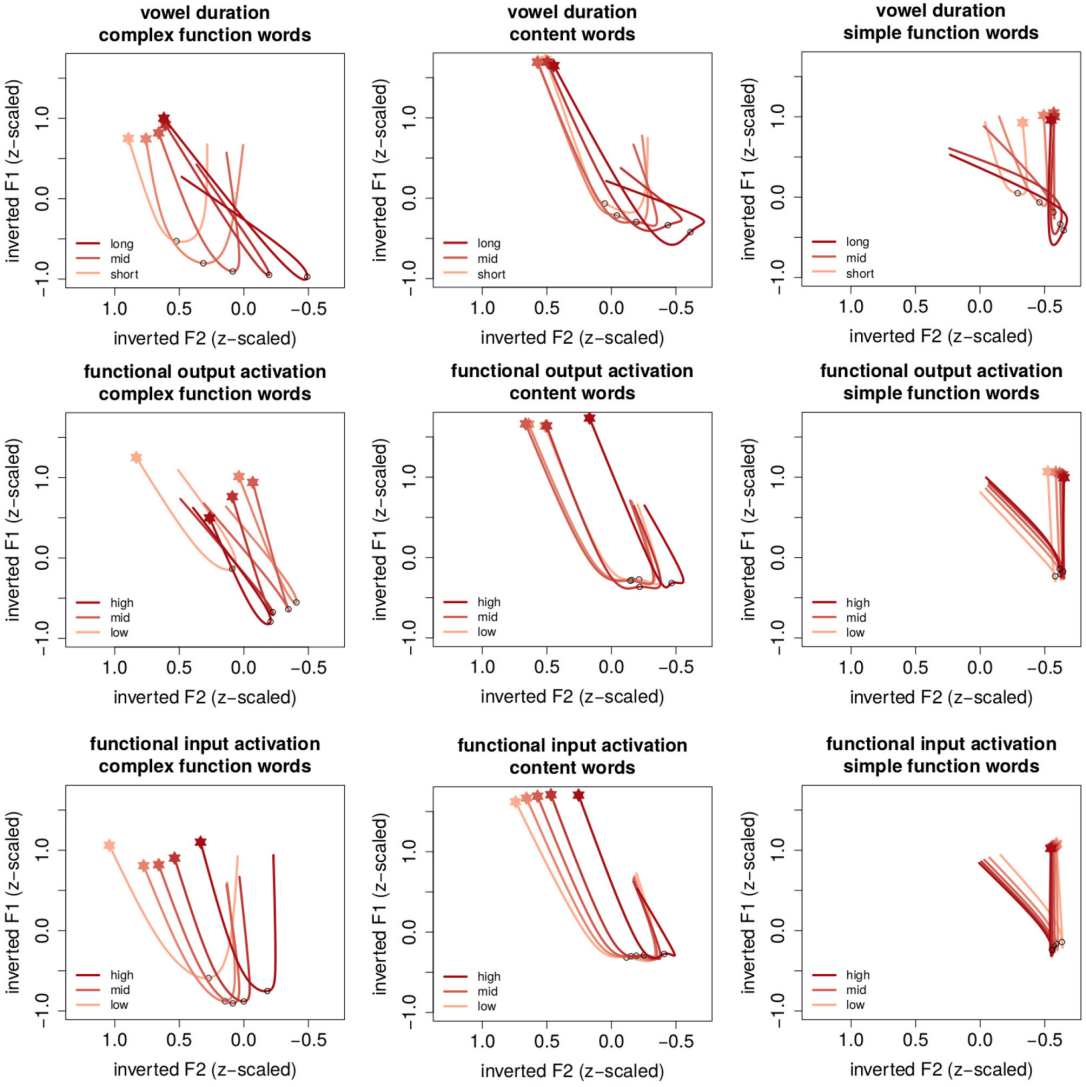
Estimated trajectories for different word classes (columns) in relation to vowel duration **(top)**, functional output activation obtained from a network with inflectional functions of [ɐ] in the output **(middle)** and functional input activation obtained from a network with inflectional functions of [ɐ] in the input **(bottom)**. The x-axes represent inverted z-scaled F2 frequencies such that the left edge points toward the front of the vowel space and the right edge points toward the back of the vowel space. Y-axes represent inverted z-scaled F1 frequencies such that the top points to the top of the vowels space and the bottom points toward the bottom of the vowel space. Shades of red represent percentiles for different predictors (optimized for color blindness). Onset of the time course is located at the filled star, the circle in the trajectory represents the center of the vowel.

Figure 3 provides a visualization of the summed effects of the models presented in Table 1 by means of estimated trajectories. The x-axes represent inverted z-scaled F2 frequencies such that the left edge points toward the front of the vowel space and the right edge points toward the back of the vowel space. Y-axes represent inverted z-scaled F1 frequencies such that the top points to the top of the vowels space and the bottom points toward the bottom of the vowel space. The onset of the trajectories is indicated with a filled star, its center with a circle. Columns represent different word classes (from left to right: content words, morphologically simple function words and morphologically complex function words). Rows represent different numeric predictors.

The onset of the formant trajectories in all three word classes is located at a high fronted position, followed by a fall. Roughly at the mid point of the vowel trajectory (indicated by the black circle), the trajectory makes a turn that results in raised positions. Focusing on the differences between word classes reveals that formant trajectories in morphologically complex function words (left column) are produced at the most fronted position; those in content words are relatively centered (mid column); the trajectories in morphologically simple function words are produced at the most retracted position (right column).

Formant trajectories further differ in their shapes. [ɐ] vowels in morphologically complex word forms have, on average, a relatively wide u-shaped trajectory, while morphologically simple function words have a very narrow trajectory. Moreover, it seems that the differences in shape between word classes is mirrored by the relative horizontal position in the vowel space (ignoring the effect of *vowel duration*): more fronted trajectories have wide trajectories than more retracted trajectories. In conclusion, we observe systematically different formant trajectories in relation to word class. These shapes are further modulated by *vowel duration* and *activation*.

Before we discuss the effects of the *vowel duration* and *activation* predictors, it will be first necessary to discuss how reduction and enhancement can be expected to be reflected in [ɐ]. Typically, reduction of vowels is reflected by more centralized formant trajectories. However, since [ɐ] is already located in the center of the vowel space in a very dense vocalic environment surrounded by [ə] and [a] in the vertical dimension and by [ɪ], [ʏ] and [ɔ] in the horizontal dimension, the specific direction enhancement will take is unclear. Enhancing [ɐ] in any direction and dimension may result in potential competition with its neighboring vowels.

To establish how enhancement and reduction are manifested in [ɐ], we shall first inspect how they are manifested in relation to hyperarticulation and hypoarticulation in long and short vowels. The top row of Figure 3 illustrates the effect of *vowel duration* (from the *functional input activation* models). Shades of red represent the 10th, 30th, 50th, 70th, and 90*th* percentile of *vowel duration* with darker shades of red representing longer vowels. Longer vowels are associated with longer formant trajectories, and lower and more retracted vocalic centers in all three word classes. This is a typical effect on the continuum between hypoarticulation and hyperarticulation associated with phonetic duration (Gay, 1978; Lindblom, 1990). Additionally these results show that longer vowels have stronger fronted offsets than shorter vowels. As a result, trajectories for longer vowels are “crossed”. How might one account for this effect? First, the offset of the trajectory tends to be located roughly in the center of the vowel space. Second, [ɐ] should not be retracted too far to the back as it may enter into a vowel space where it would compete with the mid low vowel [ɔ]. In order to apply both constraints, long [ɐ] result in narrower trajectories, even though when they are hyper articulated.

#### 4.2.3. Effects of Functional Output Activation

The effect of *functional output activation* is illustrated in the mid row of Figure 3. Higher percentiles of *functional output activation* are represented by means of darker shades of red. In morphologically complex function words, higher *functional output activation* is associated with lower, slightly more fronted positions. Comparing the effect to that of *vowel duration*, the lowering could be regarded as an enhancement effect. In content words, there is no observable effect apart from very high percentiles that are associated with more retracted positions. Finally, even though the main effect for *functional output activation* is significant in both dimensions in morphologically simple words, there are comparatively little changes across the activation continuum. In other words, *functional output activation* co-determines the [ɐ] trajectory only in morphologically complex function words.

#### 4.2.4. Effects of Functional Input Activation

Next, we turn our attention to how *functional input activation* modulates the [ɐ] trajectory. In both morphologically complex function words and content words, higher *functional input activation* is associated with stronger retracted formant trajectories. Using the effect of vowel duration as a baseline, we thus observe more enhancement under lower uncertainty, and reduction under higher uncertainty about [ɐ]. The way *functional input activation* co-determines formant trajectories points in the same way as the effect of vowel duration. The effect of *functional input activation* for content and morphologically complex function words is thus consistent in both the temporal and spectral domains.

However, in morphologically simple function words the effect seems to be reversed. Higher *functional input activation* produces slightly more fronted trajectories^7^. Since this effect is only minimal, we refrain from interpreting it to indicate reduction under lower uncertainty. Rather, we conclude that, perhaps unsurprisingly, *functional input activation* has no effect for morphologically simple words.

### 4.3. Vowel Duration

Even though we controlled for vowel duration during our investigation of formant trajectories, it is still possible that it is also correlated with *functional output activation* and *functional input activation*. Recall that Tucker et al. (2019) and Tomaschek et al. (2019) reported that lower uncertainty about inflectional functions was associated with longer phonetic duration. A Spearman's rank correlation indicated that vowel duration has a correlation of ρ = −0.01 (Pearson's *r* = −0.03) with *functional output activation* and ρ = 0.06 (Pearson's *r* = 0.07) with *functional input activation*. Thus, the correlation between our activation measures and [ɐ] duration is very small. To statistically evaluate these effects, we fitted log-transformed [ɐ] duration as a function of *functional output activation* and *functional input activation*. We performed a linear mixed-effect regression, controlling for local speaking rate and the number of segments in the word, including random intercepts for speakers and words. The model further indicated that *functional output activation* did not significantly correlate with vowel duration (β = −0.018, se = 0.04, *t* = −0.434), while with *functional input activation* did (β = 0.36, se = 0.16, *t* = 2.81). Visual inspection indicated that the difference between low and high *functional input activation* was roughly an increase of 10 ms in vowel duration. We also tested *word class* as a predictor but found no significant effect.

Thus, in the *functional output network* we did not observe a correlation between vowel duration and activation. By contrast, the *functional input network* did yield a small, but significant effect of enhancement.

## 5. Discussion

This study sought to investigate how the uncertainty associated with inflectional functions influences the phonetic characteristics of speech. It was motivated by the contradictory findings that have been reported regarding the effects of uncertainty on production in relation to paradigmatic and morphological families, where some studies found lower uncertainty to be associated with reduction (e.g., Hay, 2004; Hanique and Ernestus, 2011; Plag and Ben Hedia, 2017), whereas others reported enhancement (e.g., Kuperman et al., 2007; Schuppler et al., 2012; Cohen, 2015; Tomaschek et al., 2021). To assess the degree to which these findings reflected differing assumptions regarding word-internal structures, we followed Tucker et al. (2019) and Tomaschek et al. (2019)'s approach and sought to allow these structures to emerge naturally, in learning. We trained two two-layer networks employing two different representations of the predictive relations relevant to learning in speech production. From these we extracted network measures that we used to gauge the uncertainty associated with the inflectional functions of German word final aschwa [ɐ] (which discriminates around sixty different inflectional functions). We used these models to investigate how the inputs and outputs presented to learning networks should be implemented so as to most appropriately represent the structure of linguistic knowledge. To this end, we tested how accurately the measures of uncertainty derived from different implementations served to predict the phonetic characteristics of [ɐ] in the speech signal.

We observed that formant trajectories of [ɐ] were enhanced in relation to decreased uncertainty in those word classes that were morphologically complex. Below we discuss this finding in more detail in relation to the two questions that guided our study: (1) What is the relation between uncertainty within the context of morphological families and phonetic characteristics and how can it be explained? (2) What kind of input-output structure most appropriately represents linguistic knowledge in speech production models?

### 5.1. Effects of Word Class

Our analyses revealed that the formant trajectories of [ɐ] systematically differed between the three word classes investigated. These systematic differences emerged independently of the uncertainty measures obtained from the learning networks. Accordingly, this finding supports the assumption that fine phonetic detail is co-determined by lexical information. In phonological theories, definitions of phones and phonemes are typically based on a mixture of impressionistic judgments and theoretical considerations. These definitions thus not only ignore differences in fine phonetic detail, they also ignore potential differences that can arise from the influence of other levels of linguistic description, such as morphology or word class. By contrast, in keeping with other studies showing that the phonetic characteristics of supposedly homophonous “phones” vary systematically according to their morphological or grammatical status (e.g., Drager, 2011; Plag et al., 2017, and references in the introduction), these results raise questions about the adequacy of the “sound units” phonological theories suppose. In particular, it appears that the phonetic detail of speech signals contains fine grained difference that are far more systematic than traditional theories have tended to assume. Moreover, it appears that these differences may actually be informative about word class in communication. Studies have demonstrated that listeners are sensitive to changes at this level of phonetic detail, and that they use them not only to discriminate phonetic (e.g., Whalen, 1983; Beddor et al., 2013) but also morphological contrasts (Kemps et al., 2005; Tomaschek and Tucker, 2021). This suggests that the whole idea that speech signals comprise phonological realizations of words that are somehow analogous to orthography may be fundamentally misguided (Port and Leary, 2005; Ramscar and Port, 2016).

### 5.2. What Kind of Input-Output Structure Should Speech Production Models Employ?

Theoretically, the network simulations reported in our study were rooted in discriminative learning (Ramscar and Yarlett, 2007; Ramscar et al., 2011, 2013a,b; Ramscar, 2019, 2021b). This framework conceptualizes learning—during perception and production—as a process that serves to discriminate informative relationships between a set of cues and a set of outcomes in a cognitive system. When it comes to modeling, this in turn raises the question of how inputs (representing cognitive cues) and outputs (representing behavioral outcomes) should be implemented so as to most appropriately capture the cognitive process in question: in this case, speech production?

This question is further complicated by the fact that computational modeling inevitably constrains the way that relevant information is represented in a simple set of inputs and outputs (Bröker and Ramscar, 2020). This problem of abstraction is particularly apparent in simple two-layer networks of the kind employed here. This is because these models do not have the hidden layers that can enable multi-layer networks to learn abstractions from data. This is both a strength and a weakness. On one hand, it limits the ability of these models to discover abstract structures—such as inflectional functions—that may be present in a set of training data. On the other hand, simply because of their simplicity, they constrain modelers to utilizing input and output structures that explicitly code for the cues and outcomes that they believe to be important to the process being modeled (see Ramscar, 2021b, for a more detailed discussion of this point).

A similar point applies to most early computational models of speech production, such as Weaver++ (e.g., Roelofs, 1997) or the Spreading-Activation Theory of Retrieval (Dell, 1986; Dell et al., 2007, and follow-up models). While they did not explicitly address learning, these models were based on traditional linguistic and psycho-linguistic theories (e.g., by Fromkin, 1971, 1973; Levelt et al., 1999) that assumed an idealize speech process in which any abstractions posited by the theory had already been learned (and hence existed as discrete elements). Accordingly, in these models the ‘lexical semantics' of a word served as an input for lemma selection, which in turn served as an input for the selection of discrete morphological structures. These then activated the abstract phoneme sequences that explicitly represented the words to be pronounced. These abstract phoneme sequences, once syllabified, could then be used to compute the execution of articulatory gestures in a high dimensional acoustic-spatio-temporal space (Browman and Goldstein, 1986; Guenther, 2016; Turk and Shattuck-Hufnagel, 2020).

The *functional input network* presented in this study shares the same general conceptualization of the role semantics as traditional models. It assumes that intended meanings serve as the (main) cues to the initiation of articulations. It thus also shares with these older models the representation of articulation as the outcome of a process that is initiated semantically. Since our model is grounded in learning—which is always subject to experience—the input structure assumed in our model is less discrete. Rather than assuming that morphological functions and lexical meanings are somehow separate dimensions of experience, we assume that learning is required to separate them. That is, we assume that discriminating lexical from morphological features is a function of exposure and learning. Further, given the skewed distribution of linguistic forms, it follows that the degree to which these dimensions are discriminated in a given item or context will vary across the lexicon (Ramscar et al., 2013b).

Accordingly, many of the simplifying assumptions embodied in these earlier models make little sense in a learning model. For example, Levelt et al. (1999)'s theory assumes that “higher level” information is forgotten once it is transformed into a representation at a “lower level”. However, this is clearly inconsistent with learning, and the idea of abstraction being a product of the learning process. Rather, from a learning perspective, it is competition between cues representing information at lower levels that enables abstractions at higher levels to form. Finally, if the simplifying assumptions made in earlier models were true, there ought to be no correlation between semantic and morphological information and the phonetic characteristics. Yet, again consistent with the idea of all of this information being discriminated/shaped in learning, the present results, along with many of the other studies we have reviewed, contradict this assumption. Semantic and morphological information clearly does correlate with acoustic characteristics.

It further follows that if the cues to semantic and morphological information must be discriminated and abstracted in order to learn speech, they must play a similar role in speech production. That is, the semantic information that was discriminated into different levels of abstraction—lexical, morphological, inflectional—in learning will then serve as the cues to executed articulatory outcomes. Once again, which cues are informative about which articulations will depend on learning; and learning will be shaped by individual experience, the distributional structure of the language and context. In an actual speaker, this learning will be continuous both in time and across the lifespan (see e.g., Ramscar et al., 2014), and will be then processed by the multiple learning mechanisms contained within the complex architecture of the human brain.

By contrast—and critically—when it comes to modeling these learning processes, a great deal of this abstract information must be simplified and discretized in order to make the learning process tractable. Moreover, depending upon the goal of the modeling exercise, the goal of making the outcomes of the learning process interpretable raises further considerations. If our goal had been to emulate human performance as accurately as possible, there exists a range of more powerful models—multi-layered, deep learning networks (Graves, 2012; LeCun et al., 2015) that are far more capable of learning to capture the many complex factors that seems to drive speech and language (Hannun et al., 2014; Jozefowicz et al., 2016). However, this same complexity inevitably leads to Bonini's paradox (Bonini, 1963), in that understanding exactly how they actually learn their functions can be as challenging as understanding children's learning itself^8^.

It is in this regard, as we noted above, that the apparent shortcoming of two-layer networks can actually be an advantage. Because these simple networks lack the hidden layers that would typically be responsible for learning complex abstractions, they require that any implementation be simplified so as to include only the information thought necessary to learning. It furthermore requires that abstractions that are assumed to be necessary to this process be made explicit, and represented in the input-output structure.

Accordingly, by employing simple two-layer network models, we were able to explicitly examine the way that abstract information such as inflectional functions ought to be represented in models of articulatory learning. This was accomplished by configuring two networks with the two different input-output structures, and then testing which of them was the better predictor of phonetic characteristics. Our results showed that the activations from the network trained with inputs that included inflectional functions served to predict the phonetic characteristics of [ɐ] better than activations from the network trained on an input structure in which these functions were outputs.

One question about these models that remains to be answered is why the *functional output model* that successfully predicted phonetic characteristics in Tucker et al. (2019) and Tomaschek et al. (2019) almost failed to do so in the present study, while the *functional input model* succeeded. The data and analyses at hand only allow for speculations. One possible answer lies in the difference between the types of acoustic signals investigated in the previous studies and in the present study. Like the majority of studies investigating effects of uncertainty associated with paradigmatic families, Tucker et al. and Tomaschek et al. focused on durations. By contrast, the present study investigated a higher dimensional spectral signal. Another possible explanation may be the amount of inflectional functions under investigation. Tucker et al. focused on two inflectional functions; Tomaschek et al. investigated nine. By contrast, here, we investigated 60 different inflectional functions. It is of course impossible to draw firm conclusions from these considerations, however it seems likely that the results of these previous studies may have been particularly dependent on the specifics of their approach. It thus follows that any conclusions one might draw from this previous work will be more limited in its generalizability than those one might draw from the current study.

### 5.3. Enhancement vs. Reduction

As we noted at the outset, the results of studies of the association between the statistical characteristics of word forms within morphological and inflectional paradigms and their phonetic characteristics in speech show an inconsistent pattern. Some studies demonstrate that higher probability of words and segments is associated with phonetic enhancement (Kuperman et al., 2007; Hanique and Ernestus, 2011; Schuppler et al., 2012; Cohen, 2015; Lõo et al., 2018; Bell et al., 2019; Tomaschek et al., 2021), others find that it is associated with phonetic reduction (Hay, 2004; Hanique and Ernestus, 2011; Cohen, 2015; Ben Hedia and Plag, 2017; Plag and Ben Hedia, 2017). As we have argued, one reason why these contradictory patterns may have emerged is because these studies often disregarded how words and their paradigms are learned. Moreover, even where learning has been taken into account, they have often disregarded the assumptions one makes about the representation of linguistic knowledge and how it can influence learning (Bröker and Ramscar, 2020).

Addressing this last problem enabled us to provide a better account of our data. By taking into account how the distributional characteristics in language are learned, we were able to show that the phonetic characteristics of [ɐ] appear to be enhanced in relation to lower uncertainty associated with inflectional functions. These results support the findings within the framework of the *Paradigmatic Signal Enhancement Hypothesis* (Kuperman et al., 2007; Hanique and Ernestus, 2011; Schuppler et al., 2012; Cohen, 2015; Lõo et al., 2018; Bell et al., 2019; Tomaschek et al., 2021). Since these findings contradict the consistent effects of reduction in syntagmatic context demonstrated in the framework of the *Smooth Signal Redundancy Hypothesis* (Aylett and Turk, 2004), the question arises how the different effects in context of syntagmatic and morphological information are to be explained.

Kuperman et al. (2007) argue that enhancement in the paradigmatic context ought to be expected, because it reflects speaker confidence about the selection of a specific word form. The more confident speakers are (i.e., their speech production systems are) about a selection, the more time they can take to actually produce it. By contrast, Cohen (2015) argued that this effect should be expected for very different reasons. Arguing from within the framework of Exemplar theory, she suggests an alternative explanation: the phonetic characteristics of less frequent word forms will be shifted toward the characteristics of a competitor in the inflectional paradigm. This has the effect of reducing these less probable forms and making more probable form seem to be more enhanced.

While both explanations have their merits, it nevertheless remains the case that they are unable to fully explain all of the effects of enhancement and reduction in relation to uncertainty that have been observed. With regards to the confidence account, it is unclear why the effects of increased confidence are not observed within syntagmatic contexts (as pointed out by Cohen, 2015). With regards to the Exemplar theory account, exactly how it accounts for other word forms in the paradigm and how they contribute to systematic changes of phonetic characteristics (as demonstrated by e.g., Kuperman et al., 2007; Tomaschek et al., 2021) remains unclear.

### 5.4. The Signal-Message-Uncertainty Distinction

So how are the different influences of uncertainty on articulation in context—syntagmatic and paradigmatic—to be reconciled? It seems clear that in some sense both the *Smooth Signal Redundancy Hypothesis* and the *Paradigmatic Signal Enhancement Hypothesis* are true, at least in context. What is needed is an explanation of what this context is and how it applies. We suggest that the answer lies in the contribution of two very different aspects of speech production: The signal and the message, and the very different way that these interact with context.

Accordingly, it is important that we be clear about what it is that we mean when we talk about the “signal”. Every type of human communication is rooted in kinematic behavior. In acoustic communication, this behavior involves the movement of the articulators, the vocal cords and all other organs necessary to produce the acoustic speech signal (see Tucker and Tomaschek, forthcoming, for an overview). In another modality, say the visual modality in sign languages or gestures, it involves the movement of the body and the limbs. By “signal”, we therefore mean both the execution of kinematic behavior to create the acoustic or visual signals and the contrasts embodied in the different signals themselves, whose properties will of course vary in context.

It is important to stress that our conceptualization of speech production contrasts with the traditional, linguistic conceptualization of communication. This means that we do not assume that speaker messages convey or contain meanings. Rather, speakers produce a signal that listeners use to discriminate the meaning intended by the speakers. The discrimination process is based on a code that has been learned in much the same way as the discriminative models described above. It follows that this code serves to condition meanings onto signals: Language users learn the relationships between the world and the speech contrasts that encode their language's representation of various states of affairs in that world. To do this, they must learn to discriminate the semantic (in its broadest sense) cues to phonetic and articulatory contrasts in context. This in turn allows speakers to use these articulatory/phonetic contrasts in context to construct messages that serve to discriminate the meanings that they have learned to condition onto the same contrasts in similar contexts.

That is, in order for two speakers to have a conversation, they must share the same “source code” (Ramscar, 2019, 2021b) that underlies the language they are using. A listener uses what they have learned about the shared code to predict the messages intended by speakers. These messages will be produced by a speaker who has learned the same—or at least sufficiently the same—shared code. From this it also follows that speakers can use this code to predict when listeners have been provided sufficient cues to discriminate the intended message. In this sense, the relationship between the signal and the message is a function of the speaker's predictions about the meaning that a listener can be expected to be able to discriminate using the signal produced by the speaker in context. With this characterization of the communication process that speech serves to underpin in mind, we now turn our attention to the way these factors influence enhancement and reduction in speech production.

We propose that the different levels of uncertainty that are associated with the signal and the message are critical to explaining why the different kinds of uncertainty that occur in different contexts have such a very different effect on articulation. Moreover, we suggest that the *signal-message-uncertainty distinction* not only explains why these two different sources of uncertainty in speech lead to these apparently contradictory effects, we further suggest that once this distinction is recognized, these effects do not appear to be contradictory at all. Rather, these two different sources of uncertainty simultaneously exert a consistent, if contrastive, influence on articulation:

(1) Lower uncertainty about the message discriminated by the signal leads to reduction.
(2) Lower uncertainty about the signal leads to enhancement.

What is more, once the importance of the *signal-message-uncertainty distinction* is recognized, it becomes clear why two seemingly sensible accounts of effects of uncertainty could nevertheless appear to contradict one another.

This is because from the perspective of this distinction, (1) can be seen as a reformulation of the many insights that led to the hypotheses put forward in the information theoretic framework by Aylett and Turk (2004); Jaeger (2010), and Cohen Priva (2015). Speakers reduce, or even delete word forms or segments when they predict that listeners can discriminate an intended message in context from the signal. This means that under the wrong assumptions about uncertainty about the message, speakers might actually reduce articulations even though the correct strategy would be to enhance them. By contrast, we suggest that when speakers expect that the message will not be fully discriminated, they enhance the signal. This may occur because of the context, because they get appropriate feedback from the listener, or because they find themselves in a noisy environment, (Lindblom, 1990; Junqua, 1993; Buschmeier and Kopp, 2012; Hay et al., 2017).

At the same time, not only is (2) consistent with the present findings, it also captures the theoretical insights captured in the *Paradigmatic Signal Enhancement Hypothesis*. Moreover, in contrast to the *Paradigmatic Signal Enhancement Hypothesis*, the scope of our hypothesis is not constrained to morphological paradigms. Rather, its scope expands to predict potential enhancement effects in all instances in which a signal has to be produced in contexts where its form will be uncertain (see also Linke and Ramscar, 2020; Tomaschek et al., 2020, for enhanced variability associated with uncertainty).

Most importantly, whether a measure—be it activations based on an artificial neural network or probabilistic measures based on information theoretic considerations—operationalizes uncertainty about the signal or the message will ultimately depend on the input-output structure provided to a model—and critically, whether that structure maintains the important distinction between signals and messages. Only when the input-output structure appropriately reflects the relevant cue-outcome relations in a given process can we draw the correct conclusions from the statistical analyses involving these measures. As we have sought to show here, establishing what the appropriate input-output structure to any given process requires detailed analysis and empirical testing. Accordingly, we suggest that questions concerning the way that uncertainty about the message and uncertainty about the signal are to be modeled across the full range of contexts in which speech is produced can only be answered by detailed future research.

## 6. Conclusions

We have investigated how uncertainty in the context of inflectional paradigms is associated to phonetic enhancement and reduction of signals discriminating the corresponding inflectional functions. To do so, we trained two learning networks and extracted measures of uncertainty from them. We found that lower uncertainty is associated to phonetic enhancement—supporting work performed within the *Paradigmatic Signal Enhancement Hypothesis* framework. This is only the case when the network was trained on the cognitively appropriate input-output structure, where inputs represent the cognitive cues discriminating articulatory gestures and outputs represent the articulatory gesture at hand. We propose a distinction based on differences in *signal-vs.-message-uncertainty* to account for an apparent contradiction in previous research looking at the effects of uncertainty on the phonetic characteristics of speech.

## Data Availability Statement

The datasets presented in this study can be found in online repositories. The names of the repository/repositories and accession number(s) can be found below: https://osf.io/8jf5s/.

## Ethics Statement

Ethical review and approval was not required for the study on human participants in accordance with the local legislation and institutional requirements. The patients/participants provided their written informed consent to participate in this study.

## Author Contributions

FT conceptualized the study, retrieved the data, and performed the modeling and statistical analysis. FT and MR wrote the manuscript and designed the study. Both authors contributed to the article and approved the submitted version.

## Funding

This research was supported by a collaborative grant from the Deutsche Forschungsgemeinschaft (German Research Foundation; Research Unit FOR2373 Spoken Morphology, Project Articulation of morphologically complex words, BA 3080/3-1 and BA 3080/3-2).

## Conflict of Interest

The authors declare that the research was conducted in the absence of any commercial or financial relationships that could be construed as a potential conflict of interest.

## Publisher's Note

All claims expressed in this article are solely those of the authors and do not necessarily represent those of their affiliated organizations, or those of the publisher, the editors and the reviewers. Any product that may be evaluated in this article, or claim that may be made by its manufacturer, is not guaranteed or endorsed by the publisher.

## Footnotes

^1^The way that knowledge about input-output structures is represented in a network trained by the error-driven learning rule is neatly demonstrated by Hoppe et al. (2022).

^2^Both models stop at the phonological representation and outsource the problem of articulatory movements to theories of articulation and their computational implementations such as Articulatory Phonology/Task Dynamic framework (Browman and Goldstein, 1986; Saltzman and Kelso, 1987) or DIVA (Guenther, 2016).

^3^The algorithm Rescorla and Wagner (1972)'s implementation of the delta rule is simply the linear form of an earlier rule proposed by Widrow and Hoff (1960); Rumelhart and McCelland (1987), and this in turn is formally equivalent to the delta-rule used in connectionist networks (Sutton and Barto, 1981).

^4^This point also applies to the error-driven learning algorithms found at the heart of most connectionist/neural network model (Jordan et al., 2002), and Bayesian models of learning (e.g., Daw et al., 2008).

^5^Concurvity is the non-linear equivalent of collinearity that, when high, can render model terms uninterpretable. See the Appendix of Tomaschek et al. (2018b) and Baayen and Linke (2020) for more information on concurvity).

^6^Since main effects are already fitted by means of s(), partial tensor product smooths are used to fit the interaction between two predictors but not the main effects.

^7^When all word classes were fitted in one joint model, i.e., m4, this effect was strongly amplified such that the difference between low and high *functional input activation* was in the range of that for content and morphologically complex function words. However, comparing individual models with the joint model indicated that this amplification was most likely due to concurvity in the joint model.

^8^At present it is unclear how the complexities of learning at multiple levels of abstraction that underlie the performance of these models is to be translated into theoretical insight. This is highlighted by recent attempts to understand the performance of multiplayer networks in language processing tasks by treating them as experimental subjects (McCloskey, 1991; Linzen et al., 2016; Wilcox et al., 2018; Futrell et al., 2019).
